# Receptors on Neurons Help Shape Worm Development

**DOI:** 10.1371/journal.pbio.1001464

**Published:** 2013-01-08

**Authors:** Richard Robinson

**Affiliations:** Freelance Science Writer, Sherborn, Massachusetts, United States of America

Animal development relies on signals that trigger or inhibit growth and differentiation in precise places and at precise times, so that a new organ, say, forms “right here” instead of “over there.” The outline of that signaling process has been known for some time, with diffusible molecules released from various sites establishing a set of gradients that form an ever-more specific map of the developing body, beginning with the anterior-posterior axis and leading to location of limbs and organs.

**Figure:**
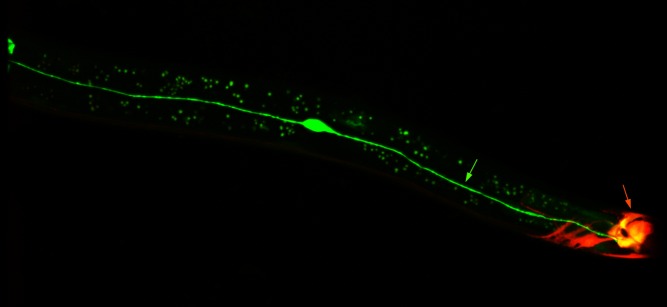
In *C. elegans*, two mid-body neurons (CANL, CANR), whose axons span the anterior-posterior axis, use their posterior axons (green arrow) to help direct head to tail patterning by a Wnt that is secreted in the posterior body (orange arrow).

A challenge for such a system is how to maintain spatial precision in the face of the increasing distances and physical changes of the growing form. In this issue of *PLOS Biology*, Katarzyna Modzelewska, Nadeem Moghal, and colleagues show that in the roundworm, neurons help meet that challenge and play a central role in specifying the location of one crucial organ, the vulva.

In the worm, the vulva arises in the middle of the body from three progenitor cells, denoted P5.p, P6.p, and P7.p. Among the many genes that are involved with proper vulval development, the authors were intrigued by *vab-8*, about which little was known except that mutations in it led to aberrant placement of the vulva along the anterior-posterior axis, and aberrant migration and axon outgrowth of a few neurons. They found that lack of the VAB-8 protein caused a variety of vulval abnormalities, including an increased frequency of developing anterior vulval progenitors, an alteration in the symmetry of the normal mid-body vulva, and formation of vulval tissue from more posterior progenitors than normal.

The *vab-8* gene was expressed in a total of ten neurons, including the two “canal associated neurons,” or CANs, which stretch the length of the head-tail (or anterior-posterior) axis. *Vab-8* gene mutation displaced the CAN cell bodies toward the head, and shortened the posterior CAN axons. When a functional *vab-8* transgene was directed only to the CAN neurons in *vab-8* mutants, it rescued the vulval phenotypes, confirming that these neurons, and not others in which *vab-8* normally is expressed, play a central role in vulval development.

Several crucial events in the development of the vulva are controlled by growth factors in the Wnt family, such as EGL-20 and CWN-1, released from cells toward the tail end. Using a marker for Wnt signaling, the authors found that, in *vab-8* mutants, there was increased Wnt activity in multiple progenitor cells. This suggested that CANs normally inhibited Wnt signaling, and that the anterior displacement and shortened axon of the mutant neurons prevented this inhibition.

Suspicion about the identity of that inhibitor fell on Ror/CAM-1, a membrane-bound tyrosine kinase that binds Wnts, including EGL-20 and CWN-1. Mutation of the *cam-1* gene induced a vulval phenotype much like that of *vab-8*, but only when the mutation affected the Wnt-binding domain, and not when it affected the interior tyrosine kinase domain. This raised the possibility that the normal function of the receptor on CANs is to sequester Wnts to inhibit signaling. Labeling of EGL-20 and CAM-1 showed similar patterns of distribution along the CAN axon, with axonal colocalization of EGL-20 being dependent on CAM-1. Thus, EGL-20 is likely enriched at CAM-1 sites, strengthening the argument that the receptor sequesters EGL-20 to reduce the total Wnt signaling reaching the vulval progenitors.

Taken together, these results paint a picture of worm development in which the two CANs play a critical role in positioning the vulva, through mopping up excess Wnt signals emanating from the posterior, reducing the local concentration sufficiently so that vulval progenitors arise and differentiate “here” and not “there.” A salient feature of this system is that it does not seem to involve signaling from the neuron itself; it is the neuron's position, not its behavior, that appears to be critical. It will now be interesting to see if the same phenomenon occurs not only elsewhere in the worm, but also elsewhere in the animal kingdom, and if so, whether neuronal signaling may also play a role in sculpting development.

**Modzelewska K, Lauritzen A, Hasenoeder S, Brown L, Georgiou J, et al. (2013) Neurons Refine the** ***Caenorhabditis elegans*** **Body Plan by Directing Axial Patterning by Wnts. doi:10.1371/journal.pbio.1001465**

